# Localized surface plasmon resonances in nanostructures to enhance nonlinear vibrational spectroscopies: towards an astonishing molecular sensitivity

**DOI:** 10.3762/bjnano.5.237

**Published:** 2014-11-28

**Authors:** Dan Lis, Francesca Cecchet

**Affiliations:** 1 Research Centre in Physics of Matter and Radiation (PMR), University of Namur (UNamur), 61 rue de Bruxelles, B-5000 Namur, Belgium

**Keywords:** coherent anti-Stokes Raman scattering (CARS), nonlinear optical spectroscopies, sum-frequency generation (SFG), surfaces plasmon resonance, vibrational spectroscopies

## Abstract

Vibrational transitions contain some of the richest fingerprints of molecules and materials, providing considerable physicochemical information. Vibrational transitions can be characterized by different spectroscopies, and alternatively by several imaging techniques enabling to reach sub-microscopic spatial resolution. In a quest to always push forward the detection limit and to lower the number of needed vibrational oscillators to get a reliable signal or imaging contrast, surface plasmon resonances (SPR) are extensively used to increase the local field close to the oscillators. Another approach is based on maximizing the collective response of the excited vibrational oscillators through molecular coherence. Both features are often naturally combined in vibrational nonlinear optical techniques. In this frame, this paper reviews the main achievements of the two most common vibrational nonlinear optical spectroscopies, namely surface-enhanced sum-frequency generation (SE-SFG) and surface-enhanced coherent anti-Stokes Raman scattering (SE-CARS). They can be considered as the nonlinear counterpart and/or combination of the linear surface-enhanced infrared absorption (SEIRA) and surface-enhanced Raman scattering (SERS) techniques, respectively, which are themselves a branching of the conventional IR and spontaneous Raman spectroscopies. Compared to their linear equivalent, those nonlinear vibrational spectroscopies have proved to reach higher sensitivity down to the single molecule level, opening the way to astonishing perspectives for molecular analysis.

## Review

### 1. Introduction – linear vibrational spectroscopies

**Figure 1:**
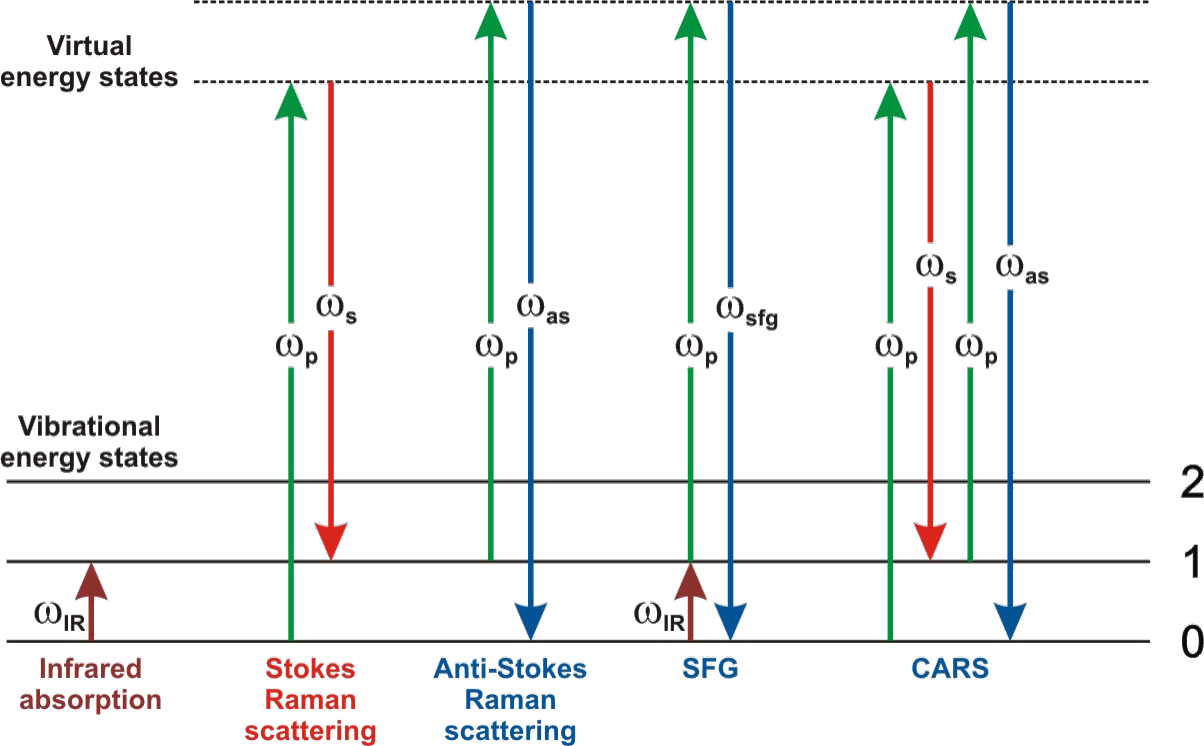
Photon diagrams for different linear and nonlinear optical processes. Solid horizontal lines are real vibrational energy states of matter, while dashed lines are virtual states. Upward pointing arrows correspond to incoming photons that lead to an excitation of matter, while downward pointing arrows correspond to emitted photons that relax the energy excitation of matter. The optical processes depicted here are linear infrared, spontaneous Stokes and anti-Stokes Raman, nonlinear sum-frequency generation, and coherent anti-Stokes Raman transitions, from left to right.

A widespread approach in molecular analysis relies on the vibrational fingerprint of matter to obtain an intrinsic chemical selectivity, and to identify specific molecules with no added labels. The two major techniques that have dominated for a long time in this field are infrared (IR) spectroscopy and spontaneous (incoherent) Raman scattering. In IR spectroscopy, IR radiation is absorbed by matter at specific frequencies matching the energy gap between vibrational states (Figure 1). Physically, it corresponds to oscillators presenting a variation of the dipole moment. In fine, the transmitted or reflected beam intensity is reduced because of the light absorption. In spontaneous Raman spectroscopy, a monochromatic visible (vis) radiation interacts with the investigated medium. Most of the illuminating light is scattered, reflected, and transmitted at the same frequency as the illumination. However, a small portion of light undergoes a change in energy during the scattering process. The energy difference between the illumination and the scattered frequencies matches a transition between vibrational states and is associated to oscillators owing a variation of polarisability: ω_Raman_ = ω_vis_ ± Δω. The shift in frequency towards the lower energy is defined as the Stokes Raman line, while the shift toward higher energy is the anti-Stokes Raman line (Figure 1).

In the frame of molecular detection at surfaces (i.e., molecular films on solid substrates), IR and Raman spectroscopies have often suffered from a lack of sensitivity due to the reduced number of oscillators in thin films on surfaces compared to bulk materials. Therefore, to make vibrational techniques more efficient for surface science purposes, many efforts have been made to increase the surface sensitivity of IR and Raman spectroscopies. For instance, from IR spectroscopy is born polarization-modulation IR reflection–absorption spectroscopy (PM-IRRAS) [1], and surface-enhanced infrared absorption (SEIRA), the latter relying on the use of surface plasmon resonances in the near-mid IR range [2]. Similarly, plasmon resonances have been used in the visible range to boost the sensitivity of Raman spectroscopy as well, in the so-called surface-enhanced Raman scattering (SERS). Indeed, spontaneous Raman scattering possesses an intrinsically weak cross section, typically lower than one Raman photon over 10^18^ [3]. However, the paths of development of SEIRA and SERS have been rather different. The success of SEIRA remains uncertain because of the limited bandwidth of plasmon resonances that makes the amplification irregular over a broad frequency window, and because of the restricted choice of efficient plasmonic materials over the extended IR range [2,4–6]. More successfully, the coupling with plasmon resonances has enhanced the Raman signal by a factor of up to 10^12^ [7]. This is possible thanks to the strong electromagnetic (EM) field amplification achieved with surface plasmon resonances, especially when the field is confined in nanometric metallic structures under the form of a localized surface plasmon resonance (LSPR) [8–16].

Up to now, a very high molecular surface sensitivity has been reached with SERS (and to a lesser extent with SEIRA), offering incredible perspectives in various fields, especially in nano-biosciences [2] in which probing tissues at the molecular level has allowed for a deeper understanding of biological properties and behaviours, and has opened the way to fascinating biomedical and biotechnological applications of single molecules and nanomaterials [7,17–20]. This success is mostly due to the electromagnetic near-field enhancement achieved thanks to more and more sophisticated nanostructures and laser sources, coupled with efficient acquisition methods.

A step forward has been achieved with nonlinear optics. Indeed, while SERS and SEIRA intrinsically possess a linear dependence on the incident laser power, nonlinear optical phenomena, instead, may benefit from a power-law relationship. Moreover, besides strict EM field intensity concerns, additional features can offer incontestable advantages, such as exploiting the molecular coherence that improves the spectroscopic yield. Many nonlinear optical processes intrinsically exhibit such coherence and are good candidates to push forward the sensitivity of vibrational spectroscopies. Within the limited number of nonlinear vibrational spectroscopies, this review will specifically focus on surface-enhanced sum-frequency generation (SE-SFG) and surface-enhanced coherent anti-Stokes Raman scattering (SE-CARS) to highlight the advantageous combination of their coherent nature with their surface-enhanced potentialities.

### 2. Nonlinear vibrational spectroscopies – SFG and CARS principles and properties

**Figure 2:**
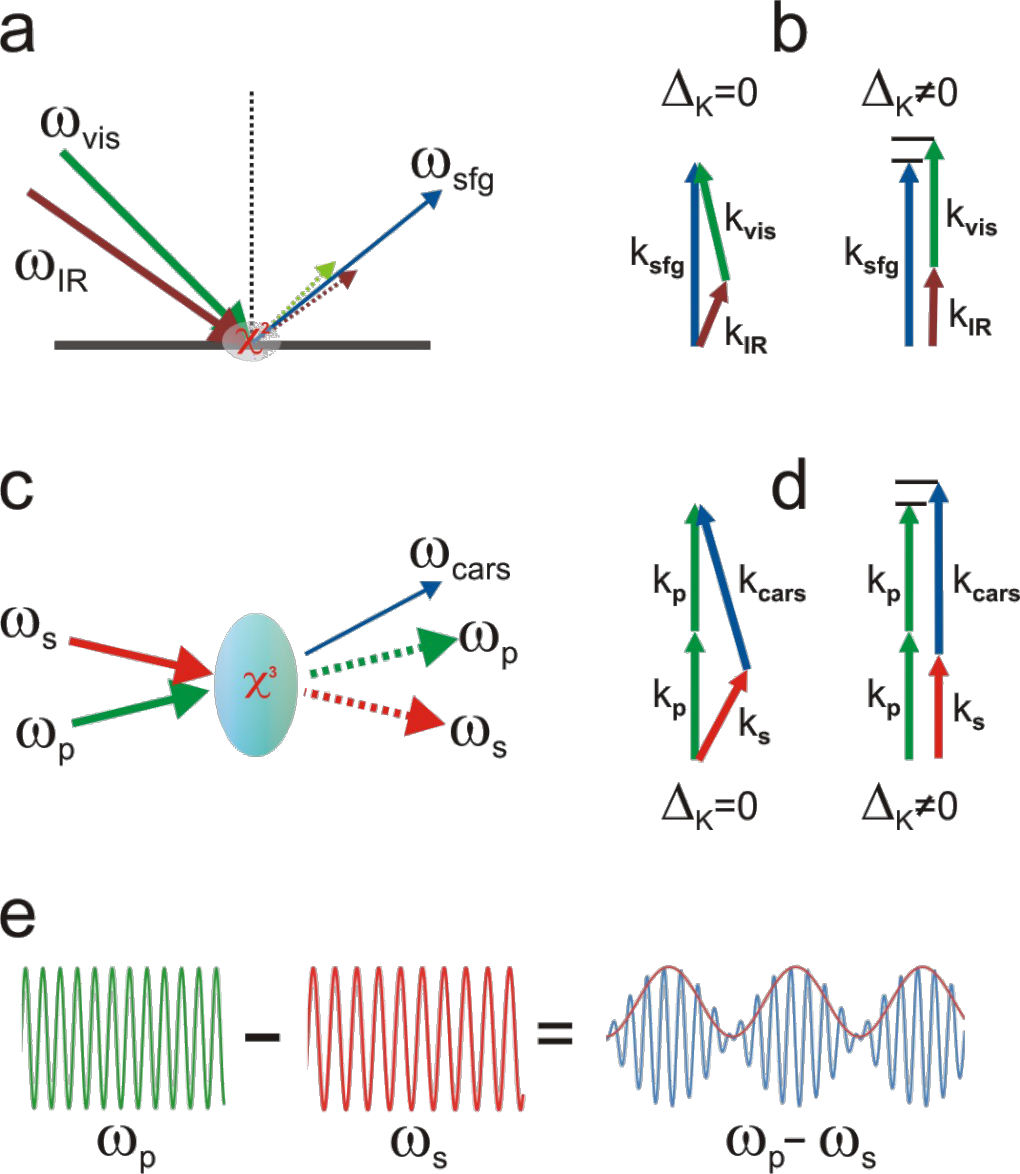
(a) Schematic representation of SFG spectroscopy in a reflection configuration on a surface. (b) The momentum conservation promotes the collinear geometry to fulfil the phase-matching condition (this condition is however relaxed for interfacial SFG). (c) Schematic representation of CARS spectroscopy performed in the bulk phase. (d) The momentum conservation requires the non-collinear geometry to be adopted to fulfil the phase-matching condition. (e) Generation of a beat frequency between the pump and the Stokes waves.

Sum-frequency generation (SFG) is a second-order nonlinear optical process in which an IR absorption is coupled to an anti-Stokes Raman process within a coherent three-wave mixing mechanism (Figure 1) [21–23]. Commonly, it is performed by focussing simultaneously one visible beam ω_vis_ and one infrared beam ω_IR_ on the interface to be probed (Figure 2a and Figure 2b). When the infrared frequency ω_IR_ matches an IR active vibrational transition, it drives all resonant oscillators in the probed volume to vibrate at that frequency. If the molecular vibration is Raman active as well, the infrared transition further interacts coherently with the visible field to drive the resonant oscillators at the sum-frequency ω_SFG_ = ω_vis_ + ω_IR_. Thanks to the very high electric field achieved with pulsed laser sources, the illuminated medium is thus polarized in a nonlinear regime and able to reemit electromagnetic waves at the SFG frequency. Being a hybrid mechanism that shares infrared and Raman activities, SFG adds up both selection rules, making it selective to a reduced number of vibrational transitions which however contain much physicochemical information about the molecular interfaces. Indeed, the analysis of the SFG signatures can provide quantitative information about the molecular orientation, and very sensitive information about the environment close to the interface from which many local properties can be deduced. Distinctly, coherent anti-Stokes Raman scattering (CARS) is a third-order optical process coupling both Stokes Raman and anti-Stokes Raman processes within a coherent four-wave mixing mechanism (Figure 1) [24–25]. It is performed by simultaneously mixing three distinct photons at two different frequencies ω_p_ and ω_s_ in the medium (see Figure 2c and Figure 2d). The pump beam ω_p_ and the Stokes beam ω_s_ interact with matter and lead to a coherent excitation of all resonant oscillators in the probed volume at the beat frequency ω_p_ − ω_s_ (Figure 2e). If this frequency matches a Raman active molecular vibration, the Stokes emission interacts coherently with the pump beam to drive the resonant oscillators at the anti-Stokes frequency ω_as_ = 2ω_p_ − ω_s_. This leads to the emission of a CARS photon at the anti-Stokes frequency. Because CARS is based on Stokes and anti-Stokes processes, it inherits the Raman selection rules [3–4]. It is noteworthy that when using the same laser source for the visible beam in SFG and for the pump beam in CARS, a given vibrational mode at ω_IR_ = ω_p_ − ω_s_ will lead to the emission of identical photons for both SFG and CARS, at a frequency ω_vis/p_ + ω_IR_ (see Figure 1).

The macroscopic polarization of the matter at the SFG and CARS frequencies results from the nonlinear interaction of the visible (ω_vis_) with the infrared (ω_IR_) fields, and the pump (ω_p_) with the Stokes (ω_s_) fields, respectively:


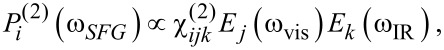



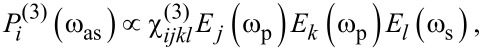


where 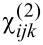 and 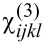
are the tensors of the second-order and third-order electrical susceptibility of the probed matter, respectively.

Two terms contribute to the measured SFG or CARS signal. The first one is referred to as the resonant term, and occurs whenever the infrared frequency ω_IR_ matches an active vibrational transition in a SFG experiment, or when the beat frequency ω_p_ − ω_s_ matches a Raman active vibration in a CARS experiment. This term gives rise to a signal corresponding to the amplitude of the vibrational mode. However, even far from any vibrational transition, a residual SFG or CARS intensity can be measured as well. This non-resonant term, which may be significant, is considered as a background signal, and origins from electronic contributions either from the substrate and/or from the molecular adsorbate [3,24–28]. Thus, the electrical susceptibility tensors are described as:


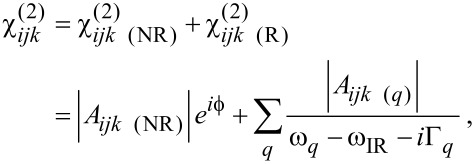



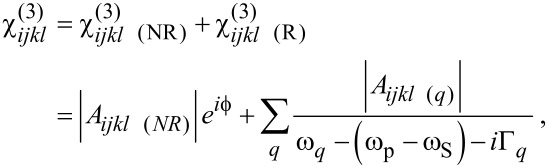


where *A_ijk_*_(_*_l_*_) (NR)_ and 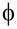 are the non-resonant amplitudes and phase; *A_ijk_*_(_*_l) _*_(_*_q_*_)_, Γ*_q_* and ω*_q_* are the oscillator strength, the damping factor and the vibrational frequency of the *q*-th vibrational mode; ω_IR_, ω_p_ and ω_S_ are the infrared, the pump and the Stokes beam frequencies. The oscillator strengths in SFG and CARS are defined as follows:


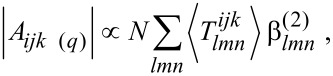



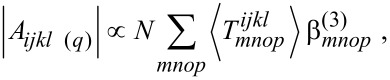


where *N* is the density number of involved individual oscillators, the brackets indicate the average over the molecular orientation distribution, *T* links the microscopic coordinates to the macroscopic ones through the Euler transformation, and β^(2)^ and β^(3)^ are the second and third order hyperpolarizability tensors, respectively. More details can be found in the literature [21,26,29–32].

The intensities of the SFG and CARS signals are given by:


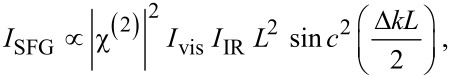



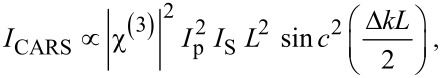


where *L* is the length over which the beams are mixed through the sample, sin *c*(*x*) = sin(*x*)/*x*, and Δ*k* is the momentum mismatch. For SFG, Δ*k*_SFG_ = *k*_SFG_ − *k*_vis_ − *k*_IR_, while for CARS Δ*k*_CARS_ = *k*_AS_ − (2k_p_ − k_S_). Since |*k*| = *n*ω/*c*, and because the energy conservation imposes that ω_AS_ − (2ω_p_ − ω_S_) = 0 and ω_SFG_ − ω_vis_ − ω_IR_ = 0, the distinct refractive indexes *n* resulting from the medium dispersion over the distinct propagating wave frequencies, promote to fulfil the phase matching condition Δ*k* = 0 in a collinear geometry (see Figure 2b and Figure 2d). Therefore, specific beam propagation angles have to be set to achieve the phase matching and ensure an optimal energy transfer from the incident beams to the nonlinear radiation over *L* [3,24]. However, for both SFG and CARS spectroscopies, this phase matching condition is relaxed under specific conditions.

Indeed, the χ^(2)^ tensor equals to zero in any centro-symmetric medium within the frame of the dipolar approximation, making the SFG photons to be generated only where the centro-symmetry is broken. Therefore, aside birefringent bulk materials, the SFG signal is commonly emitted from interfaces, such as thin films on surfaces (Figure 2a). The thickness of the emission region is thus much smaller than the coherence length (*l*_c_ = 1/Δ*k*) which is usually larger than one micron. Basically, the SFG output intensity increases nearly quadratic with the probed film thickness irrespective of the propagative angles of the laser [21]. The conservation of momentum is however applicable and determines the emission direction of the SFG in the form of a collimated beam.

Likewise, although CARS is sensitive to the bulk, it has been shown that with tightly focused laser beams, the interaction volume is rather limited and can reach dimensions smaller than the coherence length (*l*_c_ = 1/Δ*k*). The medium dispersion is thus unable to lead to a significant phase mismatch whatever the configuration [3,26,33]. This allows one to adopt either the backward- or forward-generated CARS geometry with collinear pump and Stokes beams, as largely exploited for CARS imaging with the use of commercial confocal scanning microscopes [34].

In terms of intensity, SFG scales linearly with the intensities of the infrared and of the visible beam, respectively, while CARS is proportional to the intensity of the Stokes beam and to the square of the pump beam intensities. Both of them scale with the square of the density number of oscillators, *N*^2^, in the probed volume. Interestingly, with *N* being a density number (oscillators per volume), and *L* defining the probed volume length, both SFG and CARS actually scale with the square of the oscillator surface density.

Overall, the intrinsic properties and the selection rules of SFG and CARS spectroscopies make them highly competitive with their linear counterparts regarding the sensitivity and the ability to deduce fine physicochemical information. Overall, the relative superiority of SFG and CARS derives from several factors: i) the molecular coherence that ensures a much higher conversion efficiency; ii) the emission directionality; iii) the power-law dependence of the intensities of SFG and CARS on the total incident power; iv) the square dependence of the SFG and CARS intensities on the resonant oscillators density *N*^2^; v) the blue-shifted conversion processes, which avoid any interference with fluorescence, and vi) the overall higher spatial resolution from up-frequency conversion, both lateral and for 3D layering.

Based on those arguments, on the one hand SFG, being a spectroscopy method specific to interfaces, it possesses a very high surface sensitivity and has proved to be able to provide the vibrational signature of sub-monolayer coverages [35] as well as unique orientational information about molecules at interfaces [36–47]. On the other hand, CARS has enabled rapid tissue imaging in biomedical applications. Proper image contrast can be obtained with CARS microscopy within one second and with a concentration contrast weaker than 10^6^ CH_2_ oscillators in the probed volume, corresponding to several 10^4^ lipid molecules, which are easily achieved in lipid-rich structures [3,48–49]. Nowadays, SFG and CARS techniques are established vibrational spectroscopies, overall more sensitive than conventional linear IR or spontaneous Raman spectroscopies.

To push forward the performance of both techniques, the coupling of the molecular coherence and power-law intensity dependence with the near-field enhancement from surface plasmon resonance has been initiated, and some demonstrations of an extreme sensitivity for the study and detection of surface molecules have already been given. Since both SFG and CARS mechanisms deal with visible photons, they represent ideal candidates to obtain large intensity gains from surface-enhanced (SE) mechanisms in metallic nanostructures, alike to SERS. Basically, the surface-enhanced SE-SFG and SE-CARS signals become stronger if any of the photons involved in the wave mixing process is in resonance with a SPR. Ideally, to reach the highest enhancements, all involved photons should be in resonance with the nanostructure plasmon resonances simultaneously. Similarly to SERS, this is partially achievable for low frequency vibrational bands that keep the visible and SFG radiation, or the Stokes, the pump and the CARS radiation, close to each other in frequency. However, high frequency vibrational bands spread away the different involved visible frequencies. Since it is difficult to tailor metallic nanostructures that can support very broad or multi-frequency resonances, only partial and limited plasmonic oscillations are usually obtained. Therefore, though very high theoretical enhancements have been predicted (up to 10^12^ for SE-CARS [50]), this is barely reproduced in experiments. As we will show in detail in this review, up to now, the best enhancement reported equals 10^5^ for SE-SFG, and reaches 10^7^ with SE-CARS, by comparison with the conventional SFG and CARS, respectively.

### 3 Localized surface plasmon resonance in nanostructures

As mentioned earlier, the intensity of the nonlinear conversion process can be amplified if the electric field is enhanced at least at one wave-mixing frequency, and more successfully if all fields are simultaneously enhanced. Therefore, the coupling of SFG and CARS spectroscopies with surface-enhanced fields has begun to be investigated, in particular by using propagating surface plasmon polaritons (SPP) in thin metal sheets and localized surface plasmon resonances in metal nanostructures.

**Figure 3:**
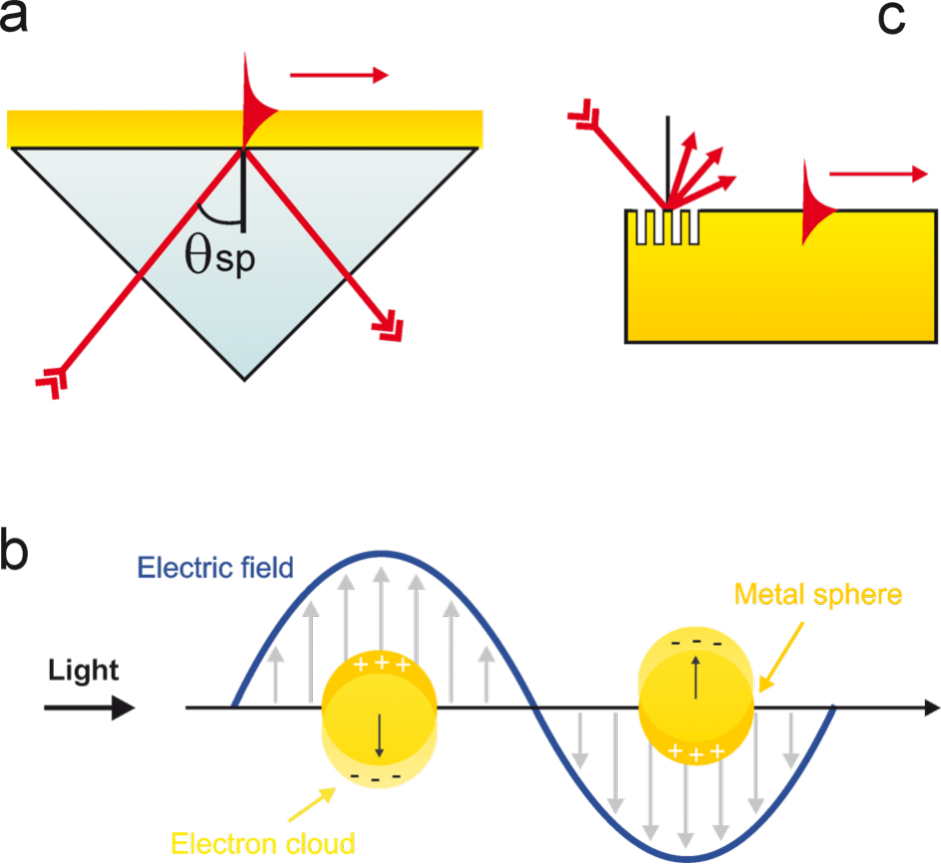
(a) Schematic representation of a SPP excitation (Kretschmann configuration) in which the plasmon mode propagates into a metal film adjacent to a prism. The incident angle should be accurately chosen so as to phase-match the momentums of the illuminated light and the SPP. (b) The excitation of a LSPR in nanoparticles confines the light energy into sub-wavelength volume, leading to strongly enhanced local electromagnetic field. (c) The excitation of a SPP can be coupled with the light diffraction on a grating.

SPP refers to the possibility of propagating an electromagnetic wave within a very thin metallic interface [51–53]. The propagating plasmon wave possesses a momentum *k*_sp(_*_x_*_)_ which is purely directed along the interface. Its excitation requires using light that has a momentum component *k_x_* equal to *k*_sp(_*_x_*_)_. Because in air *k_x_* is smaller than *k*_sp(_*_x_*_)_, a dispersive material with a higher refractive index is commonly used as coupling medium to increase the incident light wave vector and fulfil *k_x_* = *k*_sp(_*_x_*_)_. In practical devices, a prism-metal interface is employed (Figure 3a). The incident angle of the excitation light can be precisely adjusted to phase-match both wave vectors along the interface and propagate the EM field through the SPP.

While the local field amplification (denoted as *g*) can reach up to 100 times with SPP, it may goes up to 1000 times when condensing the metal sheet into nanostructures, e.g., virtually rolling it up into wires, or folding it into spheres [6]. Such strong local field amplification arises from the tridimensional shrinkage of the material into nanometric dimensions, which confines the EM field into sub-wavelength volumes (Figure 3b). Since the field is not propagating anymore but is well localized, the momentum matching condition is not required any longer. The LSPR can thus be excited by a simple reflection without considering dispersion issues. The only excitation condition is now the rigorous matching of the LSPR frequency, and eventually the use of adequate light polarization. Note that such external excitation of a LSPR can be used to funnel the SPR into a propagative mode in a metal sheet, thanks to diffraction, without considering the dispersion issue. This is currently achieved by using a metallic grating in external reflection geometry (Figure 3c).

The strong EM field enhancements *g* achieved with noble metal nanostructures have been extensively exploited in SERS. While the SERS intensity is proportional to |*g*|²·|E_0_|² = |*g*|²·*I*_0_, the specific case in which both the incident and scattered fields are in resonance with a (broad) plasmon mode makes the SERS intensity to scale up with |*g*|^4^·*I*_0_, leading to an enhancement factor of 10^12^. Accordingly, LSPR represents the most commonly used technique for state of the art surface-enhanced spectroscopies.

**Figure 4:**
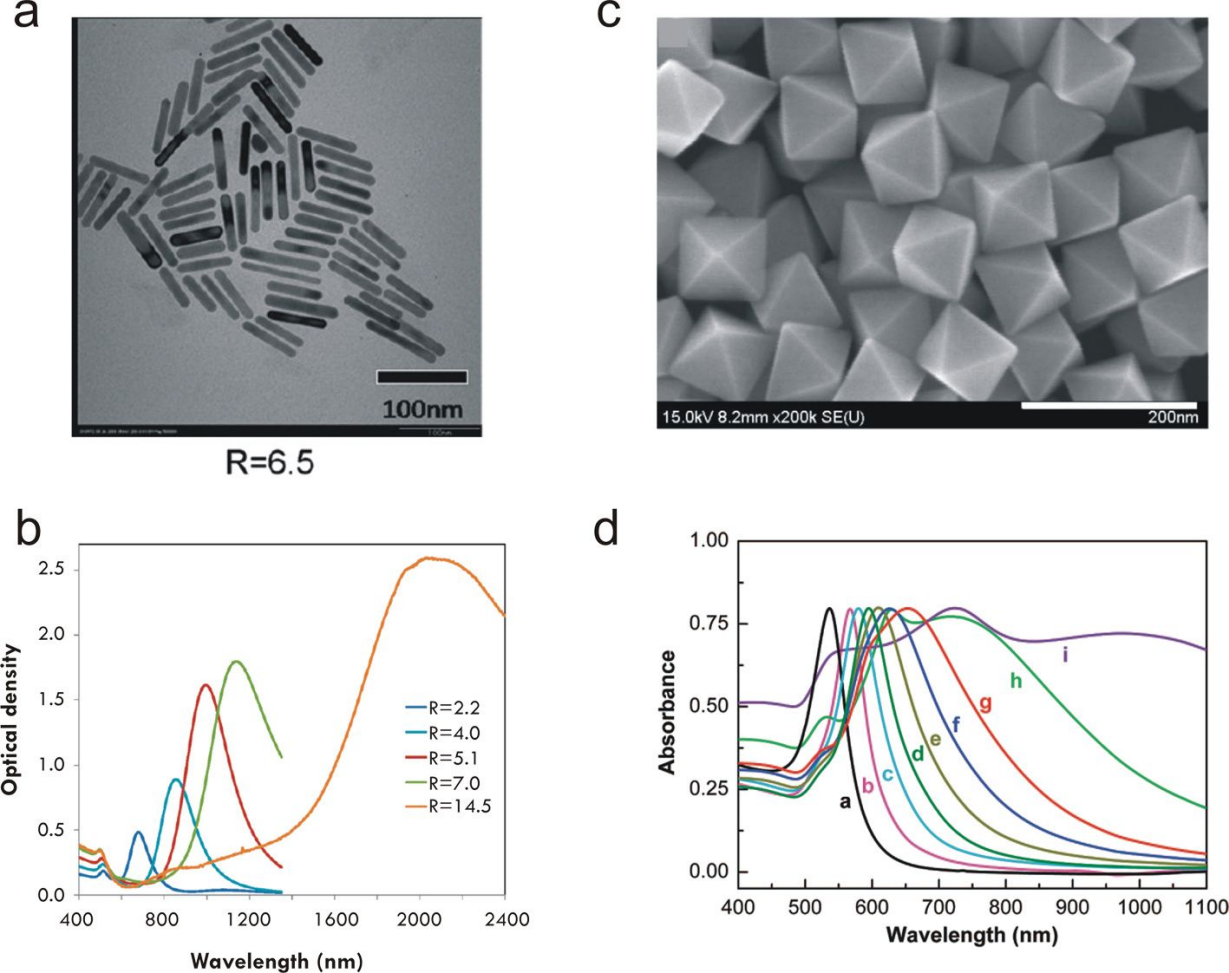
(a) TEM image of gold nanorods (AuNRs) with an aspect ratio *R* equal to 6.5. (b) UV–vis measurements showing the experimental spectra of longitudinal and transverse LSPRs for AuNRs with various aspect ratios (c) TEM image of gold octahedra with an edge length of 95 nm. (d) UV–vis absorption spectra for Au octahedra with different edge lengths (20, 50, 63, 80, 95, 110, 125, 160, and 230 nm, from curve a to curve i, respectively) dispersed in water. Figures adapted with permission from [9], Copyright 2008 American Chemical Society, and from [12], Copyright 2009 American Chemical Society.

The frequency of the LSPR spectral peak is very sensitive to the nanostructure environment through the local refractive index. Thereby, shifts of the LSPR frequency are widely used as a method for detecting molecular interaction close to the surface of the nanoparticle [54–58]. Besides, the near-field enhancement has led to a very large variety of advances in many fundamental and applied areas of science. Large boosts in the sensitivity and intensity have been reported for a very wide variety of nanoparticle shapes, dimensions and compositions, which altogether define the light absorbance, and so the LSPR spectral peak. As shown in Figure 4, depending on those parameters, the resonance frequency can be tuned over all the visible and near-infrared ranges [9–12,15]. For example, gold nanorods (Figure 4a) present two plasmon resonances, a transverse one around 515 nm which weakly depends on the aspect ratio of the rod, and a longitudinal mode which is strongly red-shifted with increasing aspect ratio (Figure 4b). Similarly, gold octahedra (Figure 4c) display a plasmon mode highly red-shifted for increasing dimensions (Figure 4d).

**Figure 5:**
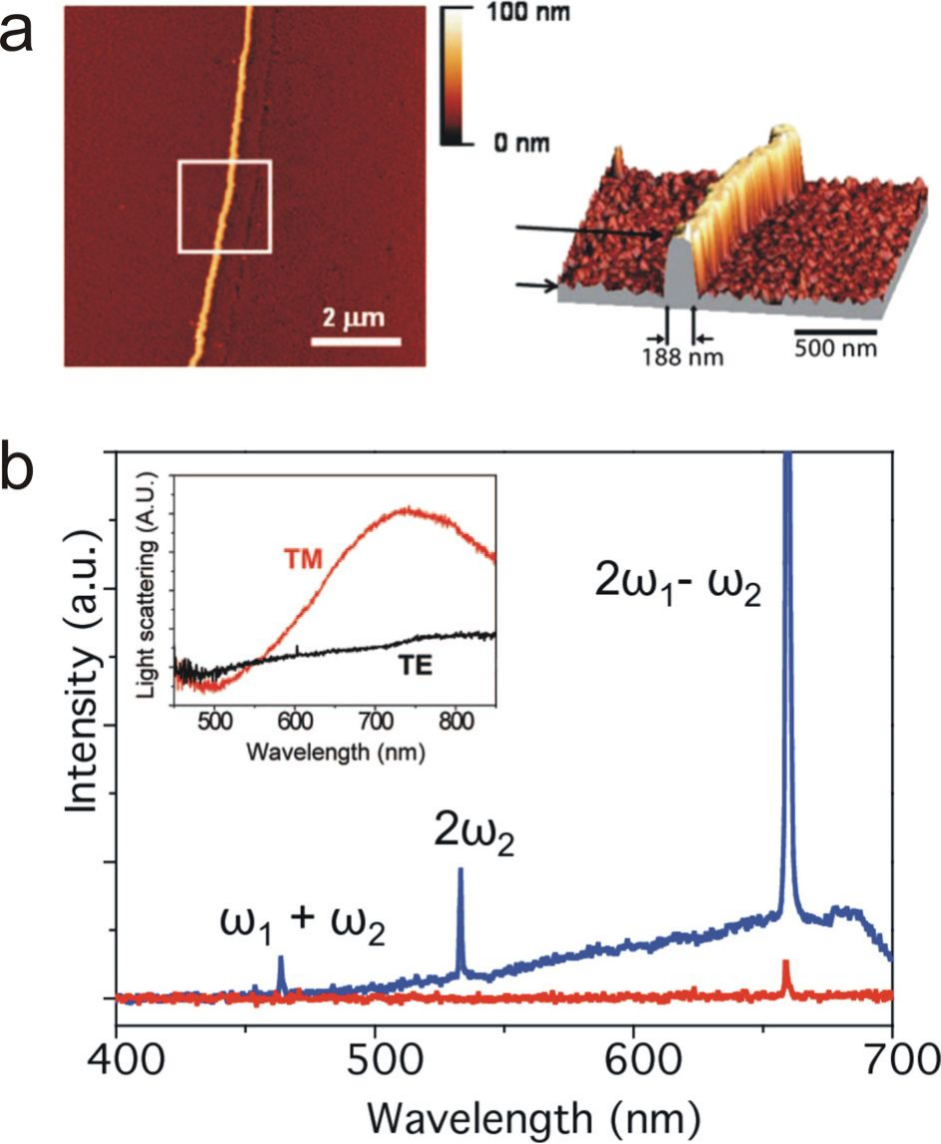
(a) AFM image of a single rectangular gold nanowire with an aspect ratio of ca. 7. (b) CARS, SFG and SHG emission from the rectangular gold nanowire. The red and blue curves correspond to a light polarization parallel and normal to the wire, respectively. The inset shows the white light scattering spectra of the wire with TM and TE polarized light. Figures adapted with permission from [32], Copyright 2010 Optical Society of America, and from [59], Copyright 2008 American Chemical Society.

Figure 5 shows the nonlinear emission of a rectangular gold nanowire illuminated simultaneously with two laser pulses at ω_1_ = 817 nm and ω_2_ = 1064 nm [32,59]. The blue spectrum plots the collected light intensity with an incident light polarization perpendicular to the wire axis, matching therefore the transverse LSPR mode of the wire. The red curve is for a light polarization parallel to the wire. The collected nonlinear response is purely non-resonant (electronic) and appears as a broad emission from the plasmon-enhanced two-photon absorption (TPA) by gold, over which three sharp peaks are observed and correspond to the nonlinear wave mixing: SFG, SHG (second harmonic generation) and CARS signals.

Interestingly, coupling nano-objects, i.e., tuning the distance between gold nanoparticles until they get in contact [60], shifts the frequency of the LSPR. For an optimal distance, i.e., when LSPR matches one of the involved laser frequencies, the nonlinear optical generation has been reported to be enhanced by four orders of magnitude because of the EM field enhancement [61].

It is important to emphasize that although using LSPR phenomena can bring a huge benefit in term of field enhancement, those effects are definitively associated with metallic surfaces, and accordingly, enhancement occurs only in the proximity of the metal surface. Because the field intensity decays exponentially, the amplification typically occurs only a few tens of nanometres away from the surface. To some extent, exploiting surface-enhanced (SE) fields limits the possibility to probe bulk materials. An alternative approach, which overcomes this limitation, consists in dispersing the nanostructures in the bulk of the medium to be probed, and use them as a contrast agent to enhance the optical signal in their surroundings, as we will discuss later.

### 4 SE-SFG spectroscopy

Sum-frequency generation emission can be increased if the electric field of the visible and/or the infrared radiation is enhanced. In principle, both SERS and SEIRA effects can contribute to the observed increase of SFG intensity, since the SFG amplification depends on the enhancement of the Raman activity, in the visible range (SERS), and/or on that of the IR activity (SEIRA) [5,62–65]. In practice, up to now, plasmon-assisted SFG enhancement has been demonstrated only with visible LSPR, due to the easier availability and development of nanostructures resonating in that range, and also due to the better amplifications obtained with plasmon-assisted SERS compared to those obtained with SEIRA [2]. It is interesting to note that such enhancement due to the LSPR excitation in the visible range indirectly enables the amplification of the signature of the infrared absorption thanks to the nonlinear wave mixing.

#### 4.1 Surface enhancement from SPP

The first age of surface-enhanced SFG spectroscopy through plasmon waves dates back to 1991 when Chen and Zhang demonstrated theoretically end experimentally the excitation of surface plasmon wave (SPW) in a silver film adsorbed on a prism and covered with a Langmuir–Blodgett (LB) film of arachidic acid [66]. The authors measured the transmitted SFG signal as a function of the Vis angle in the counter-propagating geometry, and observed the excitation of the SPW through the amplification of the non-resonant SFG signal of both the silver film and the LB monolayer. In particular, they highlighted that the SFG signal from the LB film was increased by about 25% and was angularly shifted by comparison with the pure silver film. Combining their experimental results with theoretical calculations modelling the SFG emission from the interface, the authors obtained a nonlinear polarizability coefficient of the arachidic acid molecule. These results made the authors optimistic on the future possibility to use SPWs to identify molecular adsorbates.

In 1997, Alieva et al. used a grating coated with silver (200 nm thick) to couple the excitation of a surface plasmon polariton within the metal sheet and, thus, enhance the SFG signal (as shown in Figure 3c) [67]. The grating was illuminated in external reflection and counter-propagating geometry. Although no molecular signature was recorded, the enhancement of the non-resonant SFG intensity from the metallic surface itself was mentioned to be greater than 10^4^. Later in 1999, the authors demonstrated the feasibility to use a similar plasmonic substrate to amplify the resonant SFG signals of copper phthalocyanine and fullerene films adsorbed onto the silver grating [68].

#### 4.2 Surface enhancement from porosity

In 2005, Mattei et al. observed a SFG enhancement by tuning the optical properties of silicon layers through porosity [69]. While this work does not strictly rely on plasmon excitation, tuning the electronic and optical properties of matter through morphology is an interesting alternative that, under some conditions, can lead to enhancement of the electromagnetic field at the interface. Indeed, the authors successfully amplified the SFG signal of the H-terminated porous silicon surfaces (with increasing porosity). Although they did not provide any explanation about the amplification mechanism, they were able to ascribe the result to both optical and structural effects. More interestingly, the authors studied the possibility to increase the SFG signal of H-terminated surfaces in a photonic crystal structure, namely a Fabry–Perot microcavity, made of alternating silicon layers of low and high porosity. From calculations and experiments, they predicted and verified the possibility to enhance the SFG signal up to one order of magnitude for adequate incident and SFG propagation directions. Lastly, the authors also predicted that an even better amplification could be obtained if conceiving a porous silicon photonic structure with two levels of defects, so that it could be in resonance with the visible and the SFG beams simultaneously.

#### 4.3 Surface enhancement from LSPR

Besides few examples of surface-enhanced SFG from SPP waves coupled with a prism or a grating, and from photonic structures, the most successful and promising results were certainly obtained from localized surface plasmon resonances in nanostructures.

**Figure 6:**
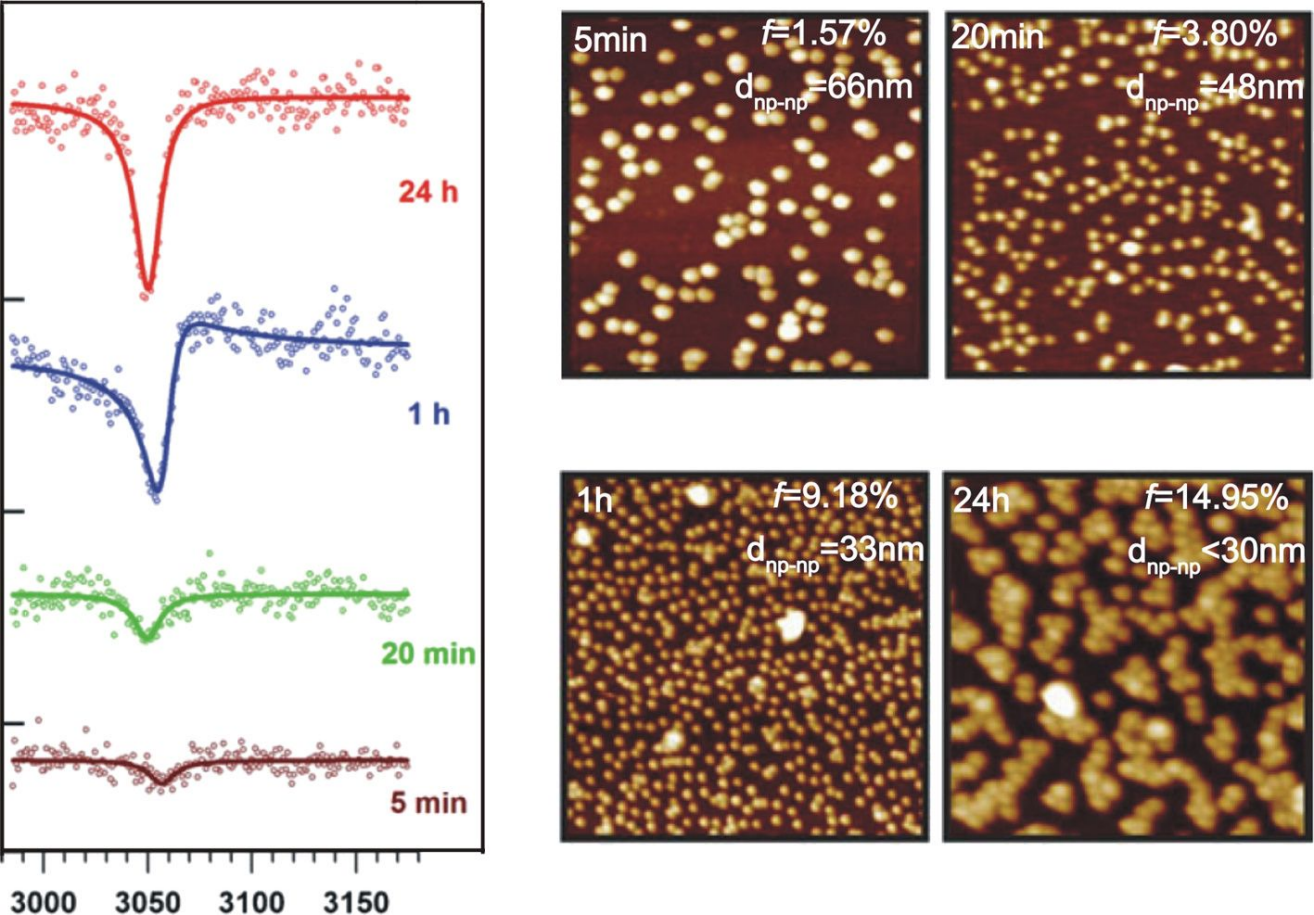
**(**Left) SFG spectra of silicon substrate immersed in a colloidal solution of gold NPs (17 nm diameters) for increasing times and subsequently functionalized with thiophenol. Depending on the immersion time, the NPs surface coverage ranged from 1.5 to 15%. (Right) Corresponding SEM images showing the progressive surface coverage with immersion time. Figure adapted with permission from [71], Copyright 2013 Springer, and from [72], Copyright 2012 Royal Society of Chemistry.

**4.3.1 Spherical nanoparticles:** In 2009, Pluchery et al. and Humbert et al. demonstrated that the vibrational SFG signature of thiophenol-functionalized gold nanoparticles (17 nm of diameter) grafted on flat silicon could be detected at a surface coverage as low as about 1% [70–71]. The SFG spectra displayed a single mode corresponding to the CH groups of the aromatic core of thiophenol at 3055 cm^−1^ (Figure 6).

The Au NPs surface coverage on a prefunctionalized aminopropyl-triethoxysilane (APTES) silicon substrate could be varied from 1.5% (5 min) to 15% (24 h) depending on the dipping time in the colloidal solution (Figure 6). When comparing the SFG signal of the thiophenol molecules chemisorbed on the NPs substrate (15% of surface coverage) with the same thiophenol SAM (self-assembled monolayer) chemisorbed on a Au(111) monocrystal, the normalized intensity enhancement was estimated to be 21. Actually, a 1.5% NPs surface coverage already led to a significant SFG vibrational signature, which underlines the remarkable sensitivity of SFG given that the intensity scale with *N*^2^. Also, thanks to this signal amplification, Humbert et al. succeeded to obtain the SFG signature of thiophenol in the fingerprint spectral range (ring deformation vibrations), where nonlinear activities are intrinsically rather weak. This was achieved by scanning the infrared between 980 and 1100 cm^−1^ with a free electron laser (CLIO) [72].

Significant amplifications of the SFG intensity from LSPR excitation were reported by other groups. In 2009, Li et al. demonstrated that a noble metal surface with a controlled roughness could lead to an important SFG enhancement [73]. To do that, a 200 nm thick silver or gold film was evaporated over polystyrene beads with various diameters (from 300 to 620 nm). The visible laser source being fixed at 532 nm, the best excitation of the LSPR mode was obtained with the 360 nm beads, no matter whether silver or gold was used as coating material. This latter fact was rather surprising since the SPR frequencies are well distinct between the two metals. However, the authors explained that, irrespective of the metal, the strong SFG enhancement was mainly due to the electromagnetic field increase at the junction between the nanospheres, with a strong dipolar contribution parallel to the substrate. This explains the necessity to use *ssp* polarization (in the order of SFG, vis, IR) to obtain a strong amplification for such dense nanoparticle layer. As a result, an octadecanethiol SAM chemisorbed on silver coated beads gave an SFG signal enhancement of 730 when considering the asymmetric stretching mode, which possesses a dipole moment mainly parallel to the surface. The enhancement of the methyl symmetric stretching was one order of magnitude weaker, according to the fact that its dipole moment change was perpendicular to the substrate, and thus penalized with regard to the directional SPR resonance lying at the nanosphere junction.

Although the amplification factor appeared much larger in this work by comparison with the result of Humbert et al., one should take into account that different normalizations were used to estimate the amplification. Indeed, in an attempt of comparison, a naive extrapolation of the surface coverage from 15% to 100% (from Humbert et al.) would increase the amplification factor from 21 to 933. This last value is much closer to the factor of 730 set by Li, although the nanoparticle size was 20 times smaller (17 nm vs 360 nm). Also, when considering the gold-coated 360 nm spheres of Li instead of the silver coated ones, the best amplification was weaker, with a factor of 190. Finally, the authors underlined that the SFG emission was strongly scattered by the surface, and that only a limited fraction of the chemisorbed molecules was precisely located at the nanosphere junctions, thereby estimating the local enhancement factor to range from 10^4^ to 10^6^. It is interesting to notice that for both gold and silver coating, the SFG vibrational modes appeared as clear peaks when amplified, while they were dip-shaped on a flat sample.

Whereas the amplification factor reflects the efficiency of the local field enhancement, spectroscopy applications are more concerned by the absolute SFG intensity that can be collected. For this sake, a common reference signal in SFG experiment is the non-resonant gold contribution in *ppp* polarization that gives an intense SFG emission. For instance, in the two above works, the intensity contrast for the strongest vibrational mode (either under the shape of a peak or a dip) never exceeded a fair 10% of that gold reference.

A hybrid configuration was studied by Tourillon et al. [74], for which the SFG spectroscopy was performed on a dense gold nanoparticles monolayer (11 nm) on a quartz prism surface. The interface was probed in the total internal reflection (TIR) configuration, in *ppp* polarization, with the visible frequency matching the LSPR of the Au NPs. According to the authors, the overall SFG signal from a dodecanethiol SAM was increased by one order of magnitude. If considering an ideal 100% surface coverage in Au NPs (which was not the case), the minimum amplification factor that can be deduced is 100. Also an important improvement of the signal-to-noise and signal-to-background ratios was obtained by comparing the TIR to the external reflection geometry. However, according to the authors, it was unclear if this enhancement resulted only from the TIR geometry, which maximized the surface EM field, or also from the excitation of a LSPR in the Au NPs. Since Tourillon et al. obtained a rather low SFG intensity when working with the same Au NPs layer functionalized with DDT, but probed in the external reflection geometry, one could believe that the contribution of the LSPR excitation was rather limited in the reported enhancement.

Nonetheless, these results underlined that the TIR configuration offers undisputable advantages for surface-enhanced SFG spectroscopy. To go further, Tourillon et al. applied this hybrid configuration to demonstrate the label-free recognition of biomolecules, namely in the case of the avidin–biocytin interaction [75]. Because SFG is sensitive to molecular order and conformation through centro-symmetry, it can address detailed molecular properties of the adlayer, which otherwise are inaccessible for most of the more conventional techniques. This enabled the authors to guarantee the specific detection of the bio-recognition and of a subsequent molecular re-ordering.

**4.3.2 Nano-antennas:** Up to now, SE-SFG has been demonstrated in only a few studies with a limited variety of nanostructures. Asides from spherical nanoparticles, the other reported nanostructure was nano-antennas. Supporting two distinct plasmon resonances, a transverse and a longitudinal mode, nano-antennas have been shown to allow large amplification of molecular signals.

**Figure 7:**
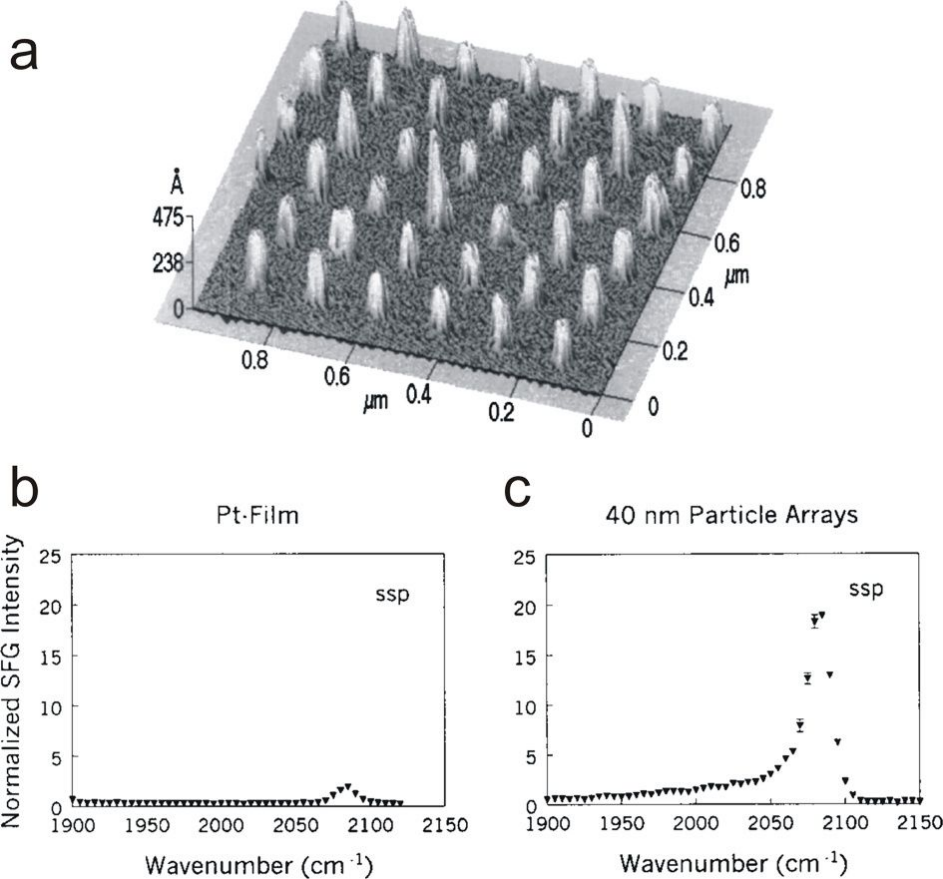
(a) AFM image of the platinum nano-antenna array, the antennas sizing 40 nm in diameter and being spaced by 150 nm. SFG spectra in *ssp* polarization of CO adsorbed on (b) a smooth Pt film and on (c) 40 nm Pt nano-antenna arrays. Figure adapted with permission from [76], Copyright 2000 AIP Publishing LLC.

In 2000, Baldelli et al. demonstrated that strong enhancements of the SFG signal could be obtained through exciting the plasmon mode in a nanopillar array [76]. They reported a significant amplification of the CO signature on an array of platinum nanopillars, prepared by electron beam lithography, and possessing uniform spacing and size (Figure 7a).

As demonstrated by the authors, the amplification was maximal in the *ssp* polarization. The calculated SFG amplification was greater than 10^4^ for a 45 nm pillar diameter and an inter-pillar distance of 150 nm (NPs surface coverage of about 5%) by comparison to the signal of CO on smooth Pt films. Note however that given the low molecular coverage, the collected SFG signal enhancement was only of a factor ca. 10 (Figure 7b,c).

**Figure 8:**
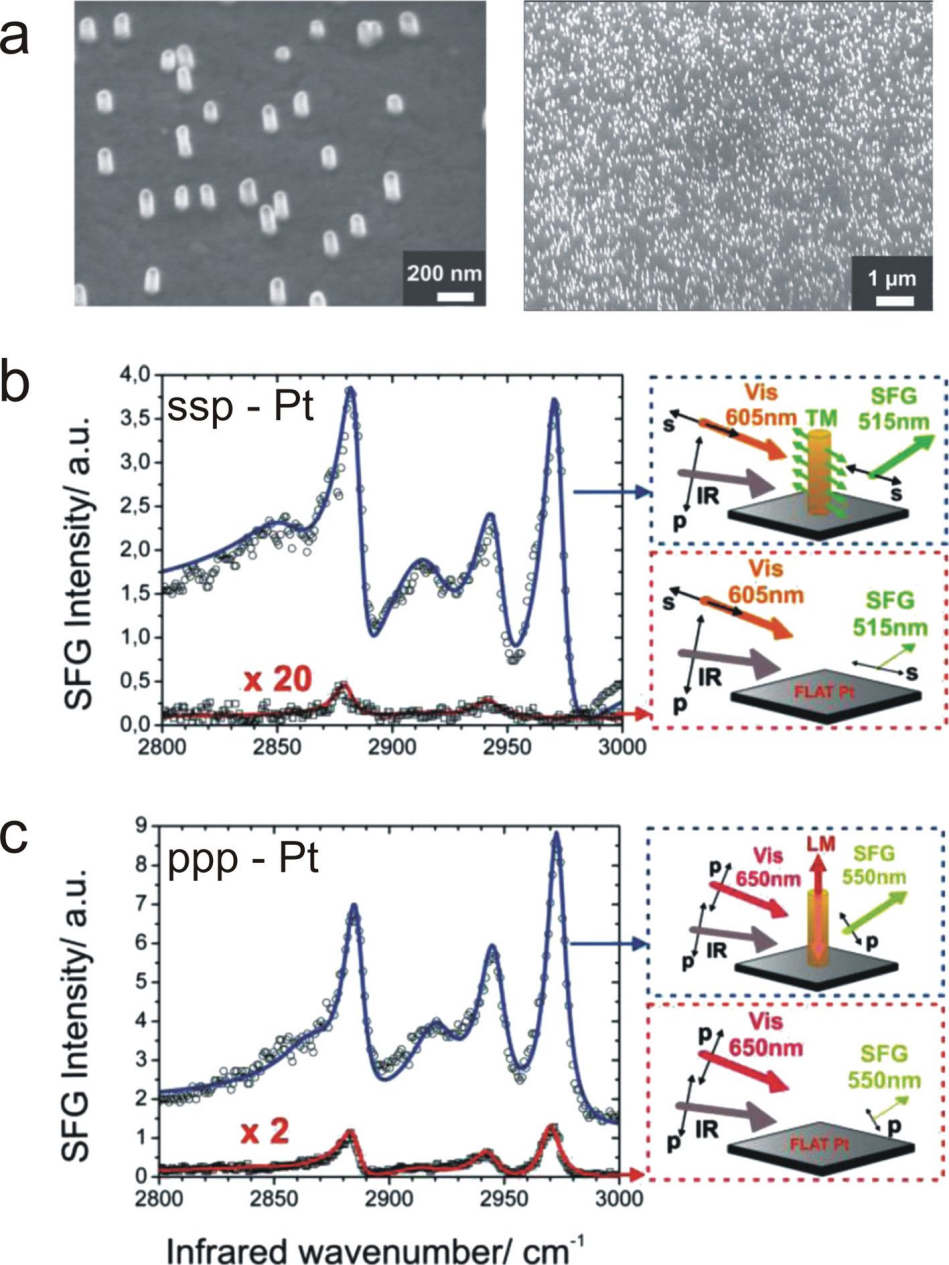
(a) Scanning electron microscopy (SEM) images of gold nano-antennas possessing an average height of 140 nm and diameter of 70 nm. SFG spectra of a dodecanethiol SAM (b) in *ssp* polarization with a selective excitation of the transverse plasmon mode and (c) in *ppp* polarization with a selective excitation of the longitudinal plasmon mode. The red curves stand for a DDT SAM chemisorbed on a flat platinum substrate, whereas the blue curves correspond to a DDT SAM over gold nano-antennas standing on flat platinum. Pictures on the right illustrate the experimental conditions in which the spectra have been recorded. Figure adapted from [77], Copyright 2013 Wiley-VCH Verlag GmbH & Co. KGaA, Weinheim.

In 2011, Lis et al. reported the enhancement of an alkanethiol SAM on randomly distributed gold nanopillars vertically-aligned on either a flat platinum or gold surface (Figure 8a) [77].

The authors achieved a selective excitation of the nanopillar LSPR modes by tuning the visible frequency over the visible range. They showed that both the transverse (TM) and the longitudinal (LM) excitation modes of the pillars could be selectively used to enhance the SFG vibrational intensity of bounded molecules. Further, because of the different EM field enhancement around the pillars when exciting either the TM or the LM mode, the authors estimated that the TM excitation could preferentially enhance the EM field at the pillar side-wall (Figure 8b), while the LM mode excitation preferentially enhanced the EM field at the pillar top (Figure 8c), leading to different areas of the nanostructured substrate to be probed. Besides, far from the LSPR excitation, the SFG signature was dominated from molecules bonded in-between the pillars on the flat substrate, which accounted for most of the sample surface. The raw SFG intensity enhancement was spectacular, and reached up to a factor of 200. By comparison to the previously used reference, that is the non-resonant gold contribution in *ppp* polarization, the resonant amplitude could reach a value of 150%. The local amplification reported by the author was several 10^3^ (10^5^ if adopting the same calculation method as used in [76])*.*

As mentioned earlier, optimizing the enhancement factor would require both the visible and SFG fields to match the plasmon resonance, simultaneously. In a SFG experiment, this is difficult since the commonly used nanostructures possess rather narrow LSPR modes, which indeed promotes multi-frequency resonances. However, the use of nanostructures that support multi-LSPR modes could potentially be a solution, as it is the case with nano-antennas, which present two modes at distinct frequencies. However the directionality of the required electric field imposes specific beam polarizations. For example, Lis et al. showed that the high frequency TM mode had to be excited with *s*-polarized SFG at 515 nm, while the low frequency LM mode had to be excited with *p*-polarized visible at 605 nm. Exciting simultaneously both modes with the visible and SFG photons would therefore impose to adopt the *sps* or polarization set which is unfavorable for the SFG signal on metals [77].

Finally, it is important to point out that in the majority of those reported SE-SFG studies, the strong amplification of the resonant features occurred with a comparable amplification of the non-resonant background. This might be explained through the surface selectivity of the SFG that makes the nanoparticle bulk contribution to be zero in the dipolar approximation. Therefore, the electronic SFG signal is limited to the surface contribution and is evenly amplified with the molecule adjacent to the nanostructure. However, care should be taken since it is known that a strong quadrupolar (and magnetic dipolar) contribution can be collected from metallic nanoparticles and could somehow lead to a SE-SFG signal as well.

### 5 SE-CARS spectroscopy

Surface-enhanced CARS performed on metallic nanostructures can be considered as the third-order nonlinear counterpart of SERS. In fact, they both rely on the Raman selection rules and benefit from the confined electromagnetic field produced by surface plasmon modes. However, as mentioned earlier, SE-CARS signal scales with the cube of the total incident intensity, and could gain an important benefit if the plasmon resonance is adequately chosen. Moreover, contrary to SERS, SE-CARS emission is coherent leading to large conversion yields over the coherence length, and ensuring a directional emission.

#### 5.1 Surface enhancement from SPP

The first experimental evidence of SE-CARS dates back to 1979 and was given by Shen et al. They performed a nonlinear mixing of four surface plasmon polariton waves that were propagated in a smooth silver film coated over a prism [78]. The surface-enhanced CARS signal was obtained from benzene molecules close to the metal interface. This work demonstrated the possibility for sub-monolayer detection at surfaces with SE-CARS.

#### 5.2 Surface enhancement from LSPR

While the possibility to obtain a higher electric field in nanostructures through LSPR drove researchers towards using nanoparticles, the first investigations dealt with bulk solutions.

**5.2.1 Bulk solutions:** In 1984, Chew et al. analytically calculated that CARS amplification of benzene molecules close to silver particles, averaged over all scattering angles, could be as large as 10^21^ [50]. Then, in 1994, Liang et al. experimentally demonstrated an enhancement of the CARS signature of benzene in the bulk phase, by adding colloidal silver nanoparticles directly into the liquid [79]. Although the phase matching condition was not fulfilled, the average particle spacing had been adjusted such as to be smaller than the coherence length, while the pump wavelength was matching the localized surface plasmon resonance of the silver NPs. In this first experimental proof, the CARS signal enhancement was limited to two orders of magnitude.

Opposite to SFG, a major drawback of CARS is the uneven amplification of the electronic contribution from the nanostructure body that can be orders of magnitude stronger than the molecular signal from the adsorbate [21,80]. To circumvent that, various experimental means have been envisioned. In 2005, Koo et al. used a polarization-sensitive CARS setup to preferentially enhance the signal coming from the analytes with respect to the background. This procedure enabled them to achieve an extremely high signal to noise ratio and single molecule sensitivity [81]. The authors used a colloidal silver mixture in which the molecular concentration was chosen so that only a single molecule was found within the probed volume, in average. They reported the SE-CARS detection of single DNA nucleotide (dAMP and dGMP, 90 pM concentration), with much stronger intensity than what could be obtained with SERS (higher by a factor of 10^3^–10^10^). Because the signal originated from single molecules, which were diffusing in and out the illuminated volume, the SE-CARS intensity was fluctuating between quantized states, proving the single molecule detection. Furthermore, this weak molecular contribution was still higher than the non-resonant contributions of water and silver particles. The authors confirmed the high sensitivity of the system with the peptide angiotensin I, as testified by the 40 times higher signal than that of SERS, while working with a molecular concentration 10^4^ times lower. This ultra-sensitive detection of non-labelled peptides achieved with SE-CARS is a brilliant example of single molecular detection and offers great perspectives for biomedical detection with very low molecular concentration threshold.

**Figure 9:**
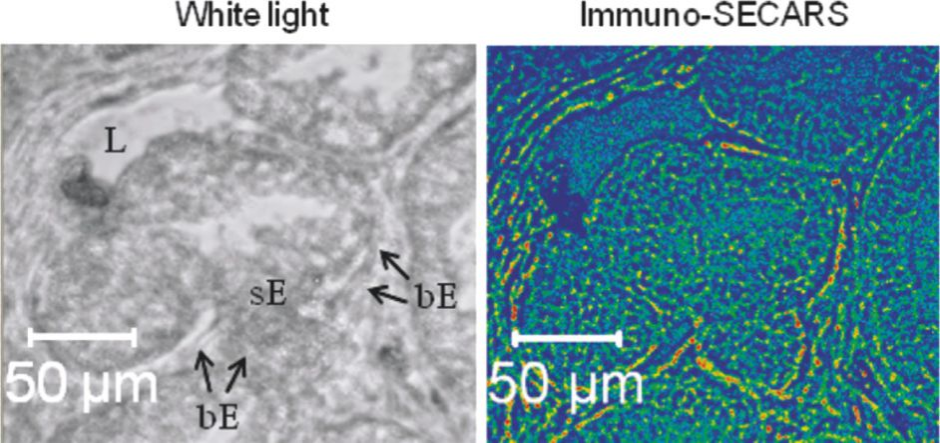
White light (left) and SECARS (right) images of prostate biopsies incubated with SERS-labeled p63-antibodies. p63 is only abundant in the p63-(+) basal epithelium (bE, arrows) but not in the p63-(−) secretory epithelium (sE) and lumen (L). Figure adapted with permission from [82], Copyright 2011 American Chemical Society.

Schlücker et al. showed in 2011 that SE-CARS could be used to obtain large scale images of tissues, for diagnostic purposes [82]. The authors demonstrated that, similarly to immunohistochemistry (IHC) that labels antibodies with dyes or enzymes to image the tissue with conventional light microscope, antibody-labeled metallic nanoshells could be used as well for accurate imaging with SE-CARS (Figure 9).

They showed the high sensitivity of the method to selectively image basal cell proteins p63 in normal prostate tissues. The authors also underlined the key advantages of immuno-SE-CARS microscopy: it enables to get very high resolution and potentially to detect different targets in the same SE-CARS experiment, by playing with the wavelength activities of different SERS labels; moreover, SE-CARS is promising for gaining time analysis, and also enables one to avoid fluorescence processes typical of biological matter, which often reduces image contrast in common immunofluorescence images. These properties promoted SE-CARS and CARS microscopy as a new tool for biomedical analysis.

Still in 2011, Namboodiri et al. investigated the SERS and SE-CARS contribution from a mixture of pyridine molecules and silver colloids [83]. They reported a SERS enhancement of pyridine up to 10^4^, while the SE-CARS signal increased barely by a factor of 10. This fact is rather surprising given that the authors took care to fulfil the phase matching condition by adopting a three-dimensional forward geometry with specific incident angles for the pump, Stokes, and probe beams, while they further delayed the pump and the probe by 1ps to reduce the non-resonant contribution, and therefore enhance the signal-to-noise ratio. The authors attributed this poor enhancement factor to the increased scattering of light due to the NPs, to the limited amount of silver clusters possessing the right shape and size to fulfil the requirements of the nonlinear coherent Raman scattering, and also to the possible destruction of those silver clusters by the high energy femtosecond-pulsed laser.

In 2014, Yamplosky et al. showed a new proof of single molecule detection threshold, as they succeeded in detecting the motion of a single bipyridylethylene (BPE) molecule in a time-resolved SE-CARS experiment [84].

**5.2.2 Surfaces:** With the perspective of a better reproducibility of the signal enhancement, different researches have been carried on solid sample devices built up with controlled parameters. Such SE-CARS spectroscopy on solid surfaces led to promising results. In 2004, Ichimura et al. succeeded to measure the SE-CARS signal from a 20 nm thick layer of adenine molecules adsorbed on isolated gold nanoparticles (60 nm) supported on a substrate [85]. The SE-CARS spectrum collected over a region of ca. 1000 µm^2^, including several tens of NPs, showed a dominant vibrational mode at 1329.5 cm^−1^, attributed to the ring breathing mode of the diazole. Further, they recorded the CARS spectrum emitted from a single nanoparticle. The local intensity was estimated to be multiplied by 2000 by comparison to the same adenine layer without nanoparticle.

Very interestingly, by using the adenine vibrational mode at 1330 cm^−1^, Hayazawa et al. succeeded to obtain a CARS vibrational mapping of a nanometric DNA network structure [86]. To do that, they used an alternative technique, namely the tip-enhanced CARS microscopy, which exploits the confined excitation of a LSPR in the extremity of a metallic tip apex to map the surface, as it is done in near-field scanning optical microscopy. This enabled to move the source of the surface-enhanced field at any place of the studied surface and achieved enhanced optical imaging at high resolution. This method also provided an enhanced CARS imaging of carbon nanotubes [86].

In 2011, Liu et al. demonstrated an advantageous configuration that coupled the use of SPP waves to generate a four-wave mixing SE-CARS signal from individual Si nanoparticles [87]. The nanoparticle was positioned over a thin gold metal film in which two distinct SSP waves counter-propagated. Although the two SPP waves were excited far from the NP itself, the propagating nature of the waves enabled the NP to generate a SE-CARS signal, free of any surface background. Also, the authors succeeded to obtain an interference pattern from the CARS generation of two adjacent NPs, proving the coherent nature of the emission. In a similar work, Wang et al. employed the Kretschmann geometry through a prism coated with a thin gold film [88]. Again, the authors used a SPP wave to locally generate a SE-CARS emission from NPs adsorbed on the gold surface. By comparison to the non-propagative wave configuration relying solely on an evanescent field, an amplification of two orders of magnitude was obtained. Finally, the authors collected the nonlinear generation on the air side of the Au film by using a lens with high numerical aperture. This enabled obtaining a SE-CARS imaging in the far-field with an emission originating exclusively from the nanostructure. Indeed, the radiation from the Au surface was prohibited in this configuration and only the dipole-like nature of the NPs radiation could lead to a light emission. In such way, the authors successfully imaged carbon nanotubes and neocyanine nanosized clusters by using a wide-field surface-sensitive SE-CARS microscope.

**Figure 10:**
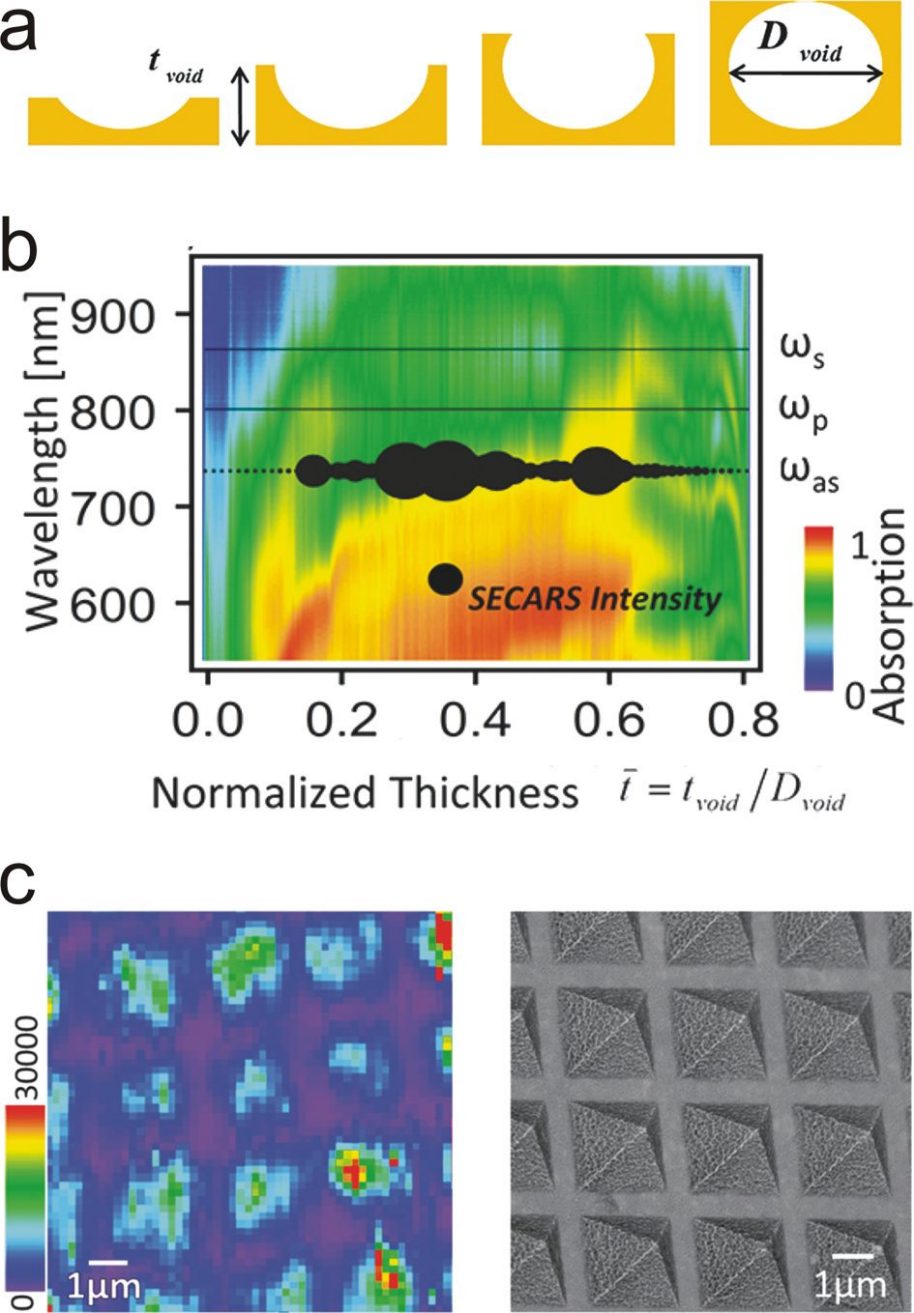
(a) Schematic representation of the gold nanovoids. Depending on the gold film thickness, the voids present various completeness levels. (b) UV–vis absorption mapping of 800 nm nanovoids as a function of their degree of completeness. For a specific thickness, the CARS and the pump frequencies can be both in resonance leading to large CARS intensities (black spot). (c) SE-CARS image of benzenethiol monolayer on the commercially available plasmonic surface Klarite (left) and corresponding SEM image showing the pyramidal pits of Klarite (right). Figure adapted with permission from [89], Copyright 2011 American Chemical Society.

As mentioned earlier, the optimum and most promising enhancement should take benefit of multi-frequency resonant devices. Those nanostructured platforms are supposed to support multiple or broad resonances, ideally matching the pump, the Stokes, and the anti-Stokes frequencies, so that CARS enhancement could be maximized. In 2011, Steuwe et al. achieved an astonishing result by using spherical gold nanovoids with intermediate depths (see Figure 10a) [89].

Figure 10b presents a mapping of the UV–vis absorption spectrum of the void structure as a function of the thickness (for a diameter of 800 nm). An evolution of the plasmonic resonance was observed with the degree of completeness of the void sphere. When the void was approximately grown up to 60% in height, the absorption was substantial for both the pump and the anti-Stokes frequencies, which led to a CARS enhancement factor of 10^5^. Besides, the authors also demonstrated that such a large enhancement factor was achievable with commercial SERS substrates (Figure 10c). They used a Klarite sample functionalized with various molecules (ferrocyanide, nitrobenzenthiol, benzenthiol). Given the broad resonances that those substrates offer, LSPR were simultaneously excited at multiple frequencies, which allowed for the very fast recording of CARS images with an estimated reproducibility over 10^4^ measurements. It is worth to mention that the authors compared the SE-CARS intensity with the conventional SERS signal (which is already 10^7^ times higher than Raman). The enhancement was 10^3^ times more intense, making SE-CARS 10^10^ times higher than conventional Raman spectroscopy on plasmonic substrates. This study demonstrated the possibility of using reproducible plasmonic surfaces able to support strong and repeatable SE-CARS intensity enhancements, and in consequence, fast vibrational imaging.

**Figure 11:**
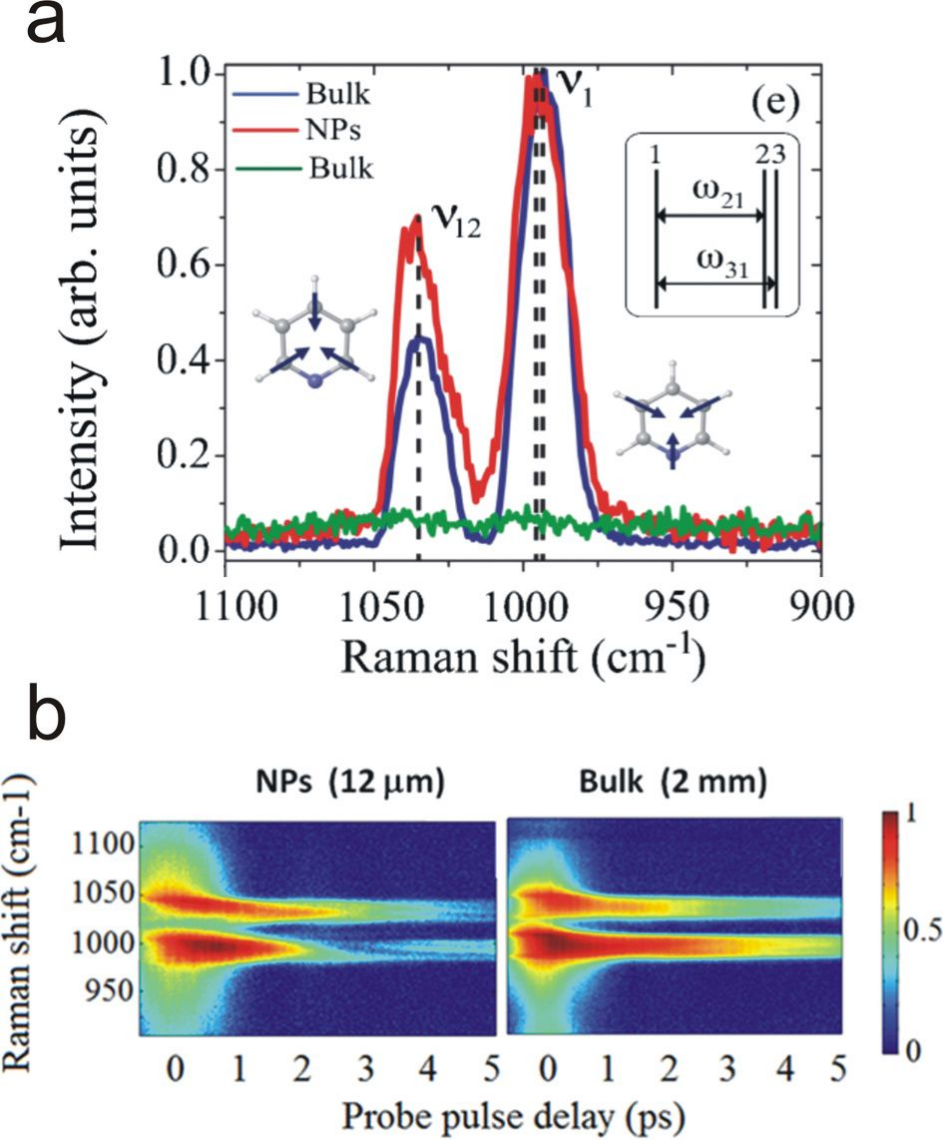
(a) SE-CARS spectrum obtained from a 12 µm thick layer of pyridine on the surface of NPs (red spectrum). The bulk CARS signal (without NPs) showed no detectable spectral features for a 12 µm thick sample (green), whereas a 2 mm thick layer gave the blue spectrum. Three vibrational modes were resolved: at 990 cm^−1^ and 997 cm^−1^ due to the ring breathing mode of free pyridine and hydrogen-bonded pyridine-water complexes, respectively; and at 1030 cm^−1^ due to the triangle mode of a mixture of both species. (b) 2D (SE)-CARS spectrograms obtained by scanning the probe pulse delay. A delay of 1 ps led to a sharp improvement of the signal-to-background ratio, while narrow band probe pulse and adequate slit in the detection set-up led to a high spectral resolution. Figure adapted with permission from [90], Copyright 2012 Nature Publishing Group.

Aside from those important achievements, investigations demonstrated that the electronic background that derives from the water and the metal could also be reduced on such kind of solid substrates. Voronine et al. used time-resolved surface-enhanced CARS, which combines delayed laser pulses of different widths, to obtain a high spectral resolution with the suppression of the non-resonant background [90]. To provide quantitative signal intensity comparisons, the SE-CARS spectroscopy was applied to a 12 µm thick pyridine–Au NPs layer (red trace in Figure 11a), while conventional CARS was done on a 2 mm thick pyridine film (blue trace in Figure 11a).

Note that a 12 µm pyridine sample did not provide any measurable CARS signal (green trace in Figure 11a). The spectra showed three vibrational modes of the free and H-bonded pyridine–water complexes. Basically, the authors used femtosecond pump and Stokes laser pulses with frequencies matching the large plasmon resonance of the aggregated gold NPs. The broadband pulses enabled to efficiently excite the vibrational coherence of the pyridine–water complex molecules in contact with gold nanoparticles. Then, they used a picosecond narrow-band probe pulse to generate the coherent anti-Stokes signal, with a very good spectral resolution. Because this probe pulse was delayed in time with the pump (1 ps), it enabled to suppress the non-resonant contribution, which possesses a shorter lifetime (Figure 11b). Finally, by comparing the peak ratio between the CARS intensity collected from the bulk pyridine (estimated coherence length of 300 µm) with the SE-CARS intensity from the 12 µm film, an enhancement factor of 10^7^ was estimated. This was 10^6^ times higher than that previously reported by Namboodiri et al. in 2011 with a similar system. This value is closer to the theoretical estimate of 10^10^ for isolated NPs [27].

Very recently, a new record for SE-CARS enhancement has been set by Zhang et al. [91], who obtained an amplification of 11 orders of magnitude compared to spontaneous Raman, by exploiting the Fano resonance generated from a nanostructure made of four discs of gold arranged in a diamond-shaped structure. This is an ultimate exciting proof of the potentialities of those coherent and nonlinear spectroscopies coupled to sophisticate and controlled plasmonic nanostructures.

## Conclusion

We have reviewed the main achievements in vibrational nonlinear optical spectroscopies enhanced through their coupling with surface plasmon waves, in particular localized surface plasmon resonances in nanostructures. It emerges that the overall development of those uncommon surface-enhanced spectroscopies is still at an early stage, as demonstrated by the small number of works in the field, and that their potential application remains very specialized. In the meantime, it is unquestionable that these works have set the basis to make SE-SFG and SE-CARS powerful and unique tools to address fundamental questions in cutting edge researches carried out in laboratories. Moreover, the constant progress in the development of stable, miniaturized, easy-to-use and cheaper laser sources, together with an optimized control and design of nanomaterials and nanostructures for plasmonics, are driving SE-SFG and SE-CARS to concrete applications, for instance in the field of bioanalytical or biomedical applications.

The advantages and limitations of SE-SFG and SE-CARS are closely related to their non-enhanced counterparts, SFG and CARS. SE-SFG, like SFG, takes advantage of the centro-symmetry selectivity which makes them specific techniques to probe the vibrational response of interfacial systems, with a high sensibility to molecular conformation and order. SFG selection rules combining both IR and Raman features, it may be advantageous to study specific vibrational modes with no interference from neighbouring modes. However, because of the reduced complexity of the fingerprint, SFG offers restricted possibilities of chemical identification. Moreover, the requirement for a vibrational mode to be strongly IR and Raman active, generally entails a large loss of intensity for most of the detected modes. Hopefully, this lack of intensity may be overcome with SE-SFG, which has demonstrated to lead to very significant amplifications. Besides, an advantageous strength of SE-SFG stands in its ability to not favour the non-resonant contribution of the substrate over the resonant vibrational contribution, and, under specific conditions, this may even improve the signal-to-noise/background ratio, thus making its surface sensitivity always better. Therefore, although SE-SFG is not yet used in practical applications or as routine techniques, it appears very promising thanks to its interfacial sensitivity and represents an extreme tool for interface investigations in fundamental science.

On the other side, SE-CARS being a third-order nonlinear process, it does not possess a specific surface sensitivity. However, this insensitivity to symmetry and the sole necessity of Raman activity usually lead to a larger SE-CARS signal over SE-SFG. This makes SE-CARS more appropriate for practical applications, such as real-time in situ label-free molecular imaging (in biomedicine for example). Also, it is overall easier to implement SE-CARS because of the requirement of laser sources exclusively tuneable in the visible range. Meanwhile, SE-CARS allows for measuring the vibrational transitions from 4000 cm^−1^ up to few 100 cm^−1^, which is extremely useful. Regarding the SE ability, scaling with the third power of the total incident intensity gives a supplementary advantage for spectroscopy performed with short laser pulses (fs) at high repetition rate (MHz), which are now widely available on the market.

Overall, while each of those spectroscopies appears to be suitable for distinct aims, there is no doubt that both SFG and CARS take large advantage in plasmon-assisted configurations. This enables to combine the high sensitivity of nonlinear processes to the physicochemical properties of matter with a unique detection threshold. Those features promote both spectroscopies as must tools to investigate fundamental properties and processes, and set the basis for further technical and analytical developments, with the purpose of practical applications, such as (bio)-molecular detection devices, or (bio)-imaging tools with improved single-molecule sensitivity.
